# Human leukocyte antigen class II-based immune risk model for recurrence evaluation in stage I–III small cell lung cancer

**DOI:** 10.1136/jitc-2021-002554

**Published:** 2021-08-06

**Authors:** Peixin Chen, Lishu Zhao, Hao Wang, Liping Zhang, Wei Zhang, Jun Zhu, Jia Yu, Sha Zhao, Wei Li, Chenglong Sun, Chunyan Wu, Yayi He, Caicun Zhou

**Affiliations:** 1 Department of Medical Oncology, Shanghai Pulmonary Hospital, Tongji University Medical School Cancer Institute, School of Medicine, Tongji University, Shanghai 200092, China; 2 Tongji University, No 1239 Siping Road, Shanghai 200433, China; 3 Department of Oncology, the Second Xiangya Hospital, Central South University, Changsha 410011, China; 4 Department of Pathology, Shanghai Pulmonary Hospital, Tongji University Medical School Cancer Institute, School of Medicine, Tongji University, Shanghai 200092, China; 5 Anhui No.2 Provincial People’s Hospital, Hefei, China

**Keywords:** immunohistochemistry, lung neoplasms, tumor microenvironment, lymphocytes, tumor-infiltrating, antigen presentation

## Abstract

**Background:**

Immunotherapy has revolutionized therapeutic patterns of small cell lung cancer (SCLC). Human leukocyte antigen class II (HLA class II) is related to antitumor immunity. However, the implications of HLA class II in SCLC remain incompletely understood.

**Materials and methods:**

We investigated the expression patterns of HLA class II on tumor cells and tumor-infiltrating lymphocytes (TILs) by immunohistochemistry staining and its association with clinical parameters, immune markers, and recurrence-free survival (RFS) in 102 patients with stage I–III SCLC with radical surgery. Additionally, an HLA class II-based immune risk model was established by least absolute shrinkage and selection operator regression. With bioinformatics methods, we investigated HLA class II-related enrichment pathways and immune infiltration landscape in SCLC.

**Results:**

HLA class II on tumor cells and TILs was positively expressed in 9 (8.8%) and 45 (44.1%) patients with SCLC, respectively. HLA class II on TILs was negatively associated with lymph node metastasis and positively correlated with programmed death-ligand 1 (PD-L1) on TILs (p<0.001) and multiple immune markers (CD3, CD4, CD8, FOXP3; p<0.001). Lymph node metastasis (OR 0.314, 95% CI 0.118 to 0.838, p=0.021) and PD-L1 on TILs (OR 3.233, 95% CI 1.051 to 9.95, p=0.041) were independent predictive factors of HLA class II on TILs. HLA class II positivity on TILs prompted a longer RFS (40.2 months, 95% CI 31.7 to 48.7 vs 28.8 months, 95% CI 21.4 to 36.3, p=0.014). HLA class II on TILs, PD-L1 on TILs, CD4, and FOXP3 were enrolled in the immune risk model, which categorized patients into high-risk and low-risk groups and had better power for predicting the recurrence than tumor stage. Pathway enrichment analyses showed that patients with high HLA class II expression demonstrated signatures of transmembrane transportation, channel activity, and neuroactive ligand–receptor interaction. High-risk SCLC patients had a higher proportion of T follicular helper cells (p=0.034) and a lower proportion of activated memory CD4-positive T cells (p=0.040) and resting dendritic cells (p=0.045) versus low-risk patients.

**Conclusions:**

HLA class II plays a crucial role in tumor immune microenvironment and recurrence prediction. This work demonstrates the prognostic and clinical values of HLA class II in patients with SCLC.

## Background

Worldwide, lung cancer has the highest mortality rate with small cell lung cancer (SCLC) making up 10%–15%.^1 2^ SCLC is known as an aggressive and recalcitrant disease in that it has fast doubling time, high growth fraction, early extensive dissemination, as well as no remarkable advances in therapeutic methods in the past three decades.^2 3^ SCLC can be classified into limited-stage SCLC (LS-SCLC) and extensive-stage SCLC (ES-SCLC), and the latter accounts for 60%–70%.^4^ ES-SCLC is principally treated with platinum-based chemotherapy with a median overall survival (OS) of approximately 9–12 months.^4^,^5^ Despite an up to 75% initial sensitivity to platinum-based chemotherapy, patients with ES-SCLC will inevitably develop resistance.^4^

Recently, immunotherapy has revolutionized cancer therapy due to promising antitumor activity and significantly improved OS.^6^ Notably, immunotherapy has shown therapy promise and has broken the situation of no superior treatment than chemotherapy in SCLC.^7 8^ IMpower133, a phase 3 clinical trial, demonstrated a better prognosis among patients who took administration of atezolizumab and chemotherapy versus those who received chemotherapy alone.^7^ Subsequent CASPIAN study obtained similar results.^9^ However, immunotherapy improves the survival of patients with SCLC by only 2 months. For patients with SCLC, little progress has been made in efficacious predictive biomarkers including programmed death-ligand 1 (PD-L1).^10^ Therefore, identification of patients who may benefit from immunotherapy is urgently needed.

Human leukocyte antigen (HLA), also called major histocompatibility complex, exerts critical roles in the interaction between tumor cells and the human immune system. HLA class I is one of the canonical HLA molecules, and the role of HLA class I-specific CD8-positive T cell responses in antitumor immune response has been well established.^11–13^ Canonically coded by HLA-DP, DQ, and DR, HLA class II is highly restricted to professional antigen-presenting cells (APCs) and participates in exogenous antigen presentation to CD4+ T cells.^11^,^14^ Nonetheless, HLA class II expression on tumor cells and immune cells can be induced by interferon (IFN)-γ.^15 16^ Evidence showed HLA class II on tumor cells affected the tumor immunogenicity, tumor migration and invasion, cancer progression, immune response, and prognosis in numerous malignancies in vitro and in vivo.^17–19^ HLA class II on tumor-infiltrating lymphocytes (TILs) was related to antigen presentation, interactions with immune cells, intracellular signaling, and prognosis of patients with cancer.^20 21^ Besides, HLA class II on TILs had a controversial correlation with tumor metastasis to lymph nodes in gastric cancer.^22^ Compared with non-small cell lung cancer (NSCLC), reduced expression of HLA class II on TILs in SCLC was considered as a form of tumor immune escape.^21^ However, the implications of HLA class II on TILs in tumor immune microenvironment and prognosis in SCLC are not completely understood.

Herein, this study aims to analyze expression patterns of HLA class II on tumor cells and TILs by immunohistochemical (IHC) staining, and its association with clinical and immune indicators and recurrence-free survival (RFS) in SCLC. Additionally, we established an HLA class II-based immune risk model by least absolute shrinkage and selection operator (LASSO) regression and investigated the enrichment pathways and immune infiltration landscape associated with HLA class II in SCLC by bioinformatics analysis.

## Materials and methods

### Patients

Altogether, 102 patients with SCLC receiving radical resection in Shanghai Pulmonary Hospital between 2014 and 2018 were selected. Clinical features were gathered from electrical medical records. Lung cancer staging was determined by the seventh tumor, node, metastases staging system.^23 24^ Surgically resected tumor samples were histologically reviewed. All patients signed informed consent.

### IHC staining

All formalin-fixed paraffin-embedded tumor tissues were dewaxed with xylene, hydrated with gradient alcohol, and washed in distilled water. And then antigen retrieval was accomplished with the target recovery solution kit (DM828 or DM829, Dako). The detailed procedure was performed as described in our previous studies.^21 25^ Experimental antibodies were rabbit antihuman HLA class II (HLA class II-DP, HLA class II-DQ, HLA class II-DR, CR3/43, Abcam), program death-1 (PD-1) (Golden bridge Zhongshan, Beijing ZM-0381), PD-L1 (E1L3N, CST # 13684S), CD3 (Dako A0452), CD4 (Dako M7310), CD8 (Dako M7103), and FOXP3 (BioLegend 320101).

### Cut-off values of HLA class II and other immune markers

The IHC staining results were independently reviewed by two pathological physicians (CW and LZhang). When the results of two independent pathologists differed, they discussed the decisions together to reach a consensus. The optimal cut-off values were determined based on survival analyses.^25 26^ X-tile software^27^ (V3.6.1) was used to generate the cut-off values of immune parameters except for PD-L1 expression on tumor cells. The cut-off points for HLA class II staining positivity on cancer cells and TILs were 5% and 25%, respectively (online supplemental figure 1A, B). The positive cut-off values of other immune markers including PD-1, PD-L1 on TILs, CD3, CD4, CD8, as well as FOXP3 were 1%, 5%, 40%, 30%, 30%, and 10%, respectively, as we demonstrated before.^28^ PD-L1 tumor proportion score ≥1% was considered positive.

10.1136/jitc-2021-002554.supp2 Supplementary data

### Establishment of immune risk model by LASSO regression

LASSO regression was applied to build an immune risk model on the basis of HLA class II. LASSO regression obtained a more refined and stable model by constructing a penalty function, compressing some coefficients, and setting some insignificant coefficients equal to zero. LASSO regression retains the advantage of subset contraction and gets good results when processing data that have complex collinearity. We obtained the LASSO risk model using EmpowerStats (V3.0, Solutions, Boston, Massachusetts, USA, R V3.4.3) based on lambda (min), that is, the lambda whose error mean was the smallest. Then X-tile software (V3.6.1) was employed to determine the optimal cut-off value that separated patients into high-risk and low-risk subgroups. The discrimination of this model was evaluated by the area under the curve (AUC) of receiver operating characteristic.

### Clinical value of HLA class II and immune risk model in SCLC

We further assessed the clinical and predictive value of HLA class II and immune risk model by downloading SCLC dataset from cBioportal Database (https://www.cbioportal.org). Samples were enrolled if complete mRNA sequencing data and survival data of patients with SCLC were accessible.

### Validation of HLA class II expression in SCLC

To further explore the expression levels of HLA class II gene and its subtypes, we downloaded RNA sequencing data of tumor tissues and normal tissues in patients with SCLC in the GEO database. And we respectively compared the expression status of HLA class II along with its 11 subtypes (HLA-DRA, HLA-DRB1, HLA-DRB4, HLA-DRB6, HLA-DPA1, HLA-DPA2, HLA-DPB1, HLA-DPB2, HLA-DQA1, HLA-DQB1, and HLA-DQB2) in tumor tissues versus normal tissues. The average expression levels of 11 subtypes constituted the total expression level of HLA class II whose best cut-off value was determined by significantly separated survival curves.

### Gene ontology terms and KEGG pathway enrichment analysis

Gene ontology (GO) and Kyoto Encyclopedia of Genes and Genomes (KEGG) enrichment analyses were performed with R (R V.3.6.3) on differentially expressed genes between high versus low HLA class II expression groups to investigate the discrepant biological pathways associated with HLA class II.^29^ GO terms consisted of three categories: molecular function (MF), cellular component (CC), and biological process (BP). Differentially expressed genes were defined at p<0.05.

### Immune infiltration analysis in SCLC by CIBERSORT

We used a public website tool CIBERSORT (https://cibersort.stanford.edu) that provided mRNA expression profiles of 22 immune cells, that is, LM22, to clarify the landscape of immune infiltration in SCLC patients with high-risk versus low-risk.^30^ The immune risk was decided by RNA expression levels of HLA class II gene, CD4, FOXP3, and CD274 encoding PD-L1. Eighty-one SCLC samples were classified into high and low-risk groups. Immune maps were depicted and correlation analyses of each immune cell were performed in both groups. Additionally, the proportion of each immune cell was compared between high-risk and low-risk groups.

### Statistical analyses

Statistical description and analyses were carried out as described in our former studies.^21 25^ The Pearson χ^2^ and Fisher exact test were applied to analyze the association of HLA class II with clinical and immune indexes. Spearman rank correlation was performed for the analysis of the linear correlation between HLA class II and other immune markers. Binary logistic regression was done to determine the independently influential factors of HLA class II. Odds ratios (OR) and 95% confidence intervals (CI) of variables were calculated in the logistic regression models. Survival data were estimated by the Kaplan-Meier method and compared with the log-rank test. The hazard ratios (HR) and 95% CI of variables were determined by Cox regression. Statistical analyses across the full text were performed on GraphPad Prism (V7.0; La Jolla, California, USA) and IBM SPSS Statistics (V22.0; IBM, Chicago, IL, USA). When the two-sided p values were less than 0.05, the differences were considered statistically significant.

## Results

### Patient characteristics

A total of 102 SCLC patients receiving radical surgery were enrolled in this study, among which 18 (17.6%) were women, 23 (22.5%) were aged ≥70 years, and 58 (56.9%) were never-smokers, as we showed previously.^28^ Among 102 patients with SCLC, 60 (58.8%) presented with stage I or II, and others with stage III. Sixty-six (64.7%) patients received adjuvant chemotherapy and 27 patients (26.5%) accepted adjuvant radiotherapy.

### HLA class II and its correlation with clinical and immune parameters

HLA class II was positively expressed on cancer cells in 9 (8.8%) and on TILs in 45 (44.1%) patients, respectively. Online supplemental table 1 listed their detailed expression status. No significant correlations were found between HLA class II on tumor cells and all clinical parameters including age, gender, smoking, T stage, N stage, distant metastasis, tumor stage, and chemotherapy. However, HLA class II on TILs was negatively associated with lymph node metastasis (p=0.002) (table 1).

10.1136/jitc-2021-002554.supp1 Supplementary data

**Table 1:** Association of HLA class II with clinical and immune parameters

Characteristics	HLA class II on tumor cells			HLA class II on TILs		
	Negative	Positive	P value	Negative	Positive	P value
Age			1.000			0.133
<70	72 (91.1%)	7 (8.9%)		41 (51.9%)	38 (48.1%)	
≥70	21 (91.3%)	2 (8.7%)		16 (69.6%)	7 (30.4%)	
Gender			0.080			0.622
Female	14 (77.9%)	4 (22.2%)		11 (61.1%)	7 (38.9%)	
Male	79 (94.1%)	5 (5.9%)		46 (54.8%)	38 (45.2%)	
Smoking status			1.000			0.065
Non-smoker	53 (91.4%)	5 (8.6%)		37 (63.8%)	21 (36.2%)	
Smoker	40 (90.9%)	4 (9.1%)		20 (45.5%)	24 (54.5%)	
T stage			1.000			0.051
1–2	79 (90.8%)	8 (9.2%)		45 (51.7%)	42 (48.3%)	
3–4	14 (93.3%)	1 (6.7%)		12 (80.0%)	3 (20.0%)	
N stage			0.494			0.002*
0	39 (88.6%)	5 (11.4%)		17 (38.6%)	27 (61.4%)	
1–2	54 (93.1%)	4 (6.9%)		40 (69.0%)	18 (31.0%)	
M stage			1.000			1.000
0	89 (90.8%)	9 (9.2%)		55 (56.1%)	43 (43.9%)	
1	4 (100.0%)	0 (0.0%)		2 (50.0%)	2 (50.0%)	
Lung cancer staging			0.733			0.066
Stage I–II	54 (90.0%)	6 (10.0%)		29 (48.3%)	31 (51.7%)	
Stage III	39 (92.9%)	3 (7.1%)		28 (66.7%)	14 (33.3%)	
PD-1 on TILs			0.028*			0.016*
Negative	59 (96.7%)	2 (3.3%)		40 (65.6%)	21 (34.4%)	
Positive	34 (82.9%)	7 (17.1%)		17 (41.5%)	24 (58.5%)	
PD-L1 on TILs			1.000			<0.001*
Negative	58 (90.6%)	6 (9.4%)		47 (73.4%)	17 (26.6%)	
Positive	35 (92.1%)	3 (7.9%)		10 (26.3%)	28 (73.7%)	
PD-L1 on tumor cells			1.000			0.318
Negative	89 (90.8%)	9 (9.2%)		56 (57.1%)	42 (42.9%)	
Positive	4 (100.0%)	0 (0.0%)		1 (25.0%)	3 (75.0%)	
CD3			0.495			<0.001*
Negative	45 (93.7%)	3 (6.3%)		38 (79.2%)	10 (20.8%)	
Positive	48 (88.9%)	6 (11.1%)		19 (35.2%)	35 (64.8%)	
CD4			0.287			<0.001*
Negative	60 (93.7%)	4 (6.3%)		45 (70.3%)	19 (29.7%)	
Positive	33 (86.8%)	5 (13.2%)		12 (31.6%)	26 (68.4%)	
CD8			0.466			<0.001*
Negative	64 (92.8%)	5 (7.2%)		48 (69.6%)	21 (30.4%)	
Positive	29 (87.9%)	4 (12.1%)		9 (27.3%)	24 (72.7%)	
FOXP3			0.268			<0.001*
Negative	63 (94.0%)	4 (6.0%)		49 (73.1%)	18 (26.9%)	
Positive	30 (85.7%)	5 (14.3%)		8 (22.9%)	27 (77.1%)	

*P<0.05 indicates statistical significance.

HLA class II, human leukocyte antigen class II; PD-1, programmed death-1; PD-L1, programmed death-ligand 1; TILs, tumor infiltrating lymphocytes.

We then analyzed the relationship between HLA class II and immune parameters by χ^2^ test and spearman rank correlation (table 1 and online supplemental table 2). HLA class II on tumor cells was only related to PD-1 on TILs (p=0.028). However, the expression status of HLA class II on TILs was associated with many immune factors, such as PD-1 on TILs (p=0.016), PD-L1 on TILs, CD3, CD4, CD8, and FOXP3 (all p<0.001, table 1). Subsequent spearman rank correlation test exhibited similar results. HLA class II expressed on TILs presented a significant linear correlation with multiple immune parameters including PD-1/PD-L1 on TILs, CD3, CD4, CD8, and FOXP3 (all p<0.001, online supplemental table 2).

To further analyze possible clinical and immune factors associated with HLA class II, we performed logistic regression analysis. Rare expression of HLA class II on tumor cells in SCLC restrains further statistical analysis. Therefore, we could only include 14 variables and investigate the risk factors for positive expression of HLA class II on TILs. Significant variables in the univariate logistic regression (lymph node metastasis, PD-1 on TILs, PD-L1 on TILs, CD3, CD4, CD8, and FOXP3) were enrolled into the multivariate logistic regression model (online supplemental table 3). After adjusting other possible confounding factors, occurrence of lymph node metastasis (OR 0.294, 95% CI 0.108 to 0.799, p=0.016) was an independent negative predictor of HLA class II on TILs. And patients with positive PD-L1 expression on TILs (OR 3.339, 95% CI 1.071 to 10.404, p=0.038) had a higher expression level of HLA class II on TILs compared with those negative (online supplemental table 3).

### Association of HLA class II and immune parameters with RFS

A total of 102 patients with SCLC were included in the RFS analysis. Fifty-six patients (54.9%) relapsed by the end of follow-up, and 49 (87.5%) of 56 patients relapsed within 2 years after surgery.

**Figure 1:**
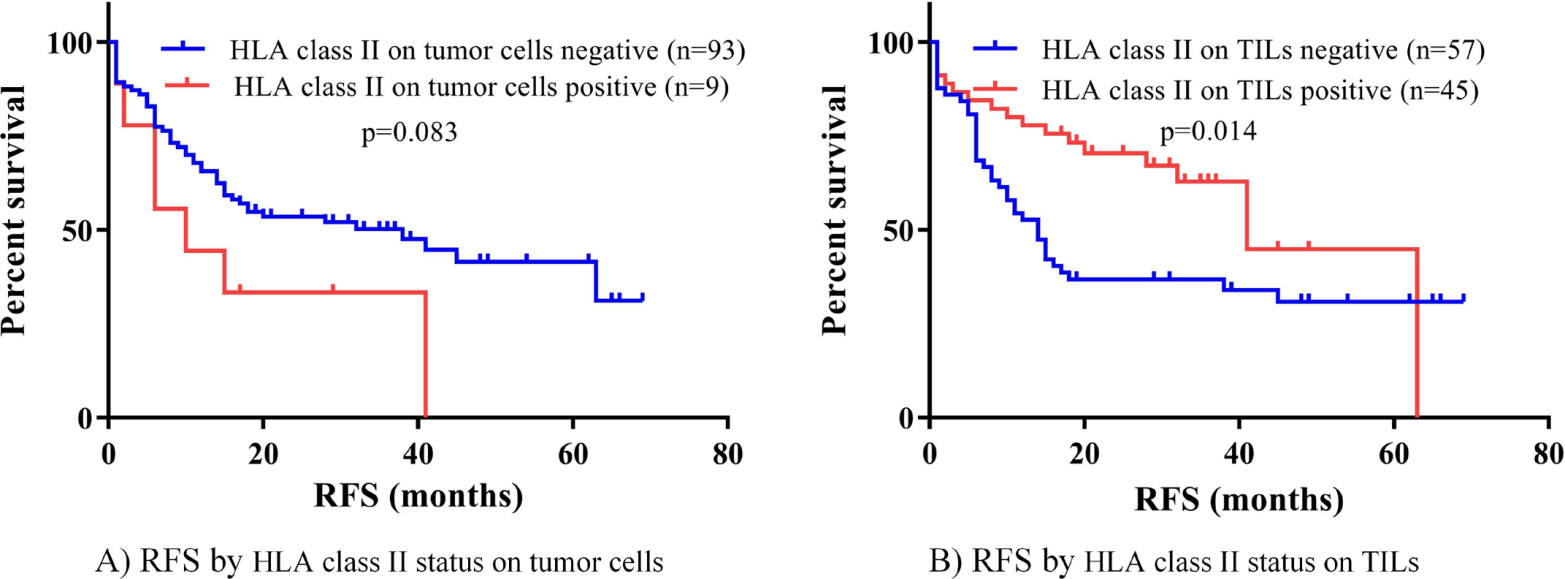
Survival analysis by HLA class II on tumor cells and TILs. HLA class II, human leukocyte antigen class II; RFS, recurrence-free survival; TILs, tumor infiltrating lymphocytes.

We first analyzed the association of HLA class II expression with RFS in these SCLC patients with the Kaplan-Meier method and log-rank test. The status of HLA class II expression on tumor cells had no significant difference in RFS (p=0.083) (figure 1A). Nonetheless, patients with positive HLA class II expression on TILs had a significantly longer RFS than those negative (40.2 months, 95% CI 31.7 to 48.7 months vs 28.8 months, 95% CI 21.4 to 36.3 months, p=0.014; figure 1B). The median RFS for patients with positive and negative HLA class II expression on TILs was 41 months (95% CI 29.1 to 53.0) and 14 months (95% CI 9.9 to 18.1), respectively.

We then enrolled HLA class II and other immune factors in the univariate Cox regression model to select possible prognostic indicators. RFS was related to the status of HLA class II expressed on TILs (HR 0.507, 95% CI 0.288 to 0.893, p=0.019) rather than tumor cells (table 2). As our previous studies have shown,^28^ RFS was also correlated with PD-L1 on TILs, and CD3, CD4, CD8, and FOXP3 on immune cells but not with PD-1 on TILs and PD-L1 on tumor cells (table 2).

**Table 2:** Univariate Cox regression analysis for recurrence-free survival

Variables	HR	95% CI	P value
HLA class II on TILs (negative vs positive)	0.507	0.288 to 0.893	0.019*
HLA class II on tumor cells (negative vs positive)	1.964	0.885 to 4.355	0.097
PD-1 on TILs (negative vs positive)^28^	0.602	0.342 to 1.059	0.078
PD-L1 on TILs (negative vs positive)^28^	0.417	0.223 to 0.779	0.006*
PD-L1 on tumor cells (negative vs positive)	0.815	0.198 to 3.347	0.776
FOXP3 (negative vs positive)^28^	0.376	0.194 to 0.730	0.004*
CD3 (negative vs positive)^28^	0.480	0.281 to 0.820	0.007*
CD4 (negative vs positive)^28^	0.450	0.245 to 0.825	0.010*
CD8 (negative vs positive)^28^	0.400	0.206 to 0.776	0.007*

*P<0.05 indicates statistical significance.

HLA class II, human leukocyte antigen class II; PD-1, programmed death-1; PD-L1, programmed death-ligand 1; TILs, tumor infiltrating lymphocytes.

### Establishment of HLA class II-based immune risk model by LASSO regression

**Figure 2:**
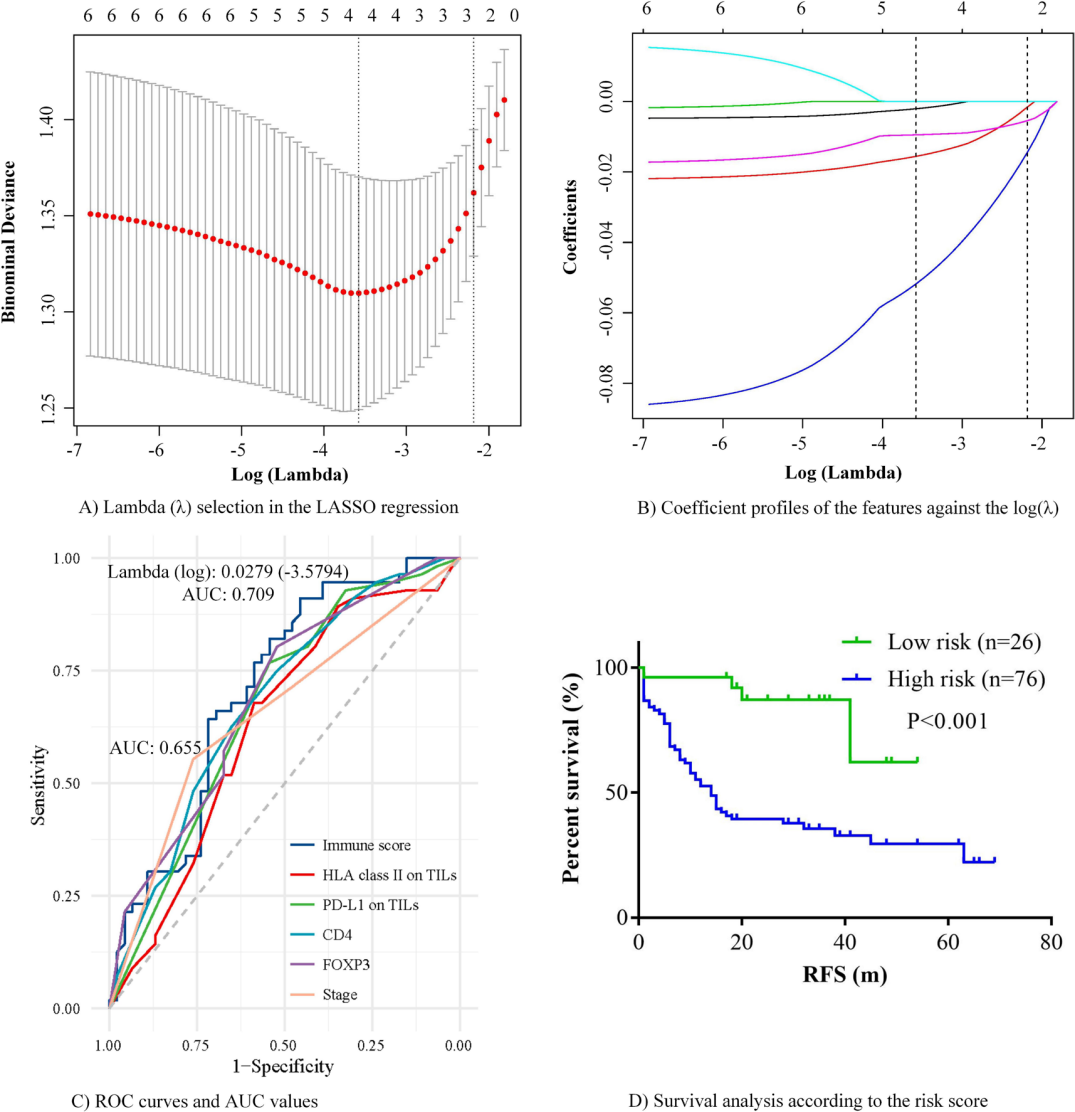
Establishment of immune risk model in SCLC. (A) The lambda (λ) selection process in the LASSO regression. Two immune risk models were obtained based on lambda.min (left dotted line) and lambda.1se (right dotted line). (B) LASSO coefficient profiles of each variable against the log(λ). The optimal immune risk model was achieved based on the lambda.min (left dotted line, 0.0279), and it enrolled four variables: HLA class II on TILs, PD-L1 on TILs, FOXP3, and CD4. (C) ROC curves and AUC values of immune risk model, single immune factors and tumor stage. The AUC value of immune risk score (0.709) exceeded that of tumor stage (0.655) and single immune factors (all <0.70). (D) Survival analysis of immune risk score. AUC, area under the curve; HLA class II, human leukocyte antigen class II; LASSO, least absolute shrinkage and selection operator; PD-L1, programmed death-ligand 1; ROC, receiver operating characteristic; SCLC, small cell lung cancer; TILs, tumor-infiltrating lymphocytes.

The results of the univariate Cox regression indicated that six immune indicators (HLA class II on TILs, PD-L1 on TILs, CD3, CD4, CD8, and FOXP3) were significantly associated with RFS in patients with SCLC. Given that spearman’s correlation analysis revealed that HLA class II on TILs had a modest linear correlation with other five immune factors, the traditional multivariate Cox regression is not applicable. Under this circumstance, we constructed an immune risk model by LASSO regression to comprehensively analyze the effects of immune indicators on the prognosis. After performing LASSO regression, we obtained two immune risk models. One is based on lambda.min corresponding to the minimum mean error and the second is on lambda.1se, that is, the maximum lambda corresponds to the minimum mean error within one standard deviation (figure 2A). Figure 2B showed the corresponding relationship between coefficients of each variable and the lambda. Based on the lambda.min (0.0279), we obtained the optimal risk model with an AUC of 0.709, which was superior to single immune markers and even tumor stage (AUC, 0.655) in terms of prediction efficiency (figure 2C). We eliminated less important factors CD3 and CD8 due to their coefficients were zero. The final LASSO formula was as follows: immune score=–(0.00206×HLA class II on TILs)–(0.01558×PD-L1 on TILs)–(0.05179×FOXP3)–(0.00947×CD4). Then we applied X-tile to determine the optimal cut-off value that separated patients into high-risk and low-risk groups. Survival analysis demonstrated that RFS in high-risk and low-risk patients with SCLC significantly differed (p<0.001) (figure 2D).

### Clinical value of HLA class II and immune risk model in SCLC

We further explored the clinical prognostic value of HLA class II and immune risk model in SCLC from the transcriptional level. In the public dataset,^31^ 77 SCLC patients with survival data were enrolled and divided into high-expression and low-expression groups based on the expression level of HLA class II. OS curves of two groups were significantly separated (p=0.016; online supplemental figure 2A). Additionally, patients with SCLC were also categorized into high-risk and low-risk groups based on RNA expression levels of four genes (HLA class II, CD274 encoding PD-L1, FOXP3, CD4). The Kaplan-Meier curve showed that high-risk patients presented a significantly poorer OS (p=0.0013) (online supplemental figure 2B).

10.1136/jitc-2021-002554.supp3 Supplementary data

### Validation of HLA class II expression in SCLC

To further explore the expression levels of HLA class II gene and its subtypes in SCLC, we downloaded RNA sequencing data of tumor and normal tissues in patients with SCLC in the GEO database. In the GSE43346 dataset, SCLC patients had a lower HLA class II expression level in comparison with normal tissues (p=0.043) (online supplemental figure 3A). For the HLA class II subtypes, we demonstrated that HLA-DRA (p=0.046), HLA-DRB1 (p=0.010), HLA-DRB4 (p=0.017), HLA-DRB6 (p=0.004), HLA-DPB2 (p=0.0005), and HLA-DQB1 (p=0.007) of tumor tissues were lower than that of normal tissues in SCLC patients, while HLA-DPA1, HLA-DPA2, HLA-DPB1, HLA-DQA1, and HLA-DQB2 were all not (p>0.05) (online supplemental figure 3B, C).

10.1136/jitc-2021-002554.supp4 Supplementary data

### GO and KEGG enrichment analysis of HLA class II expression

**Figure 3:**
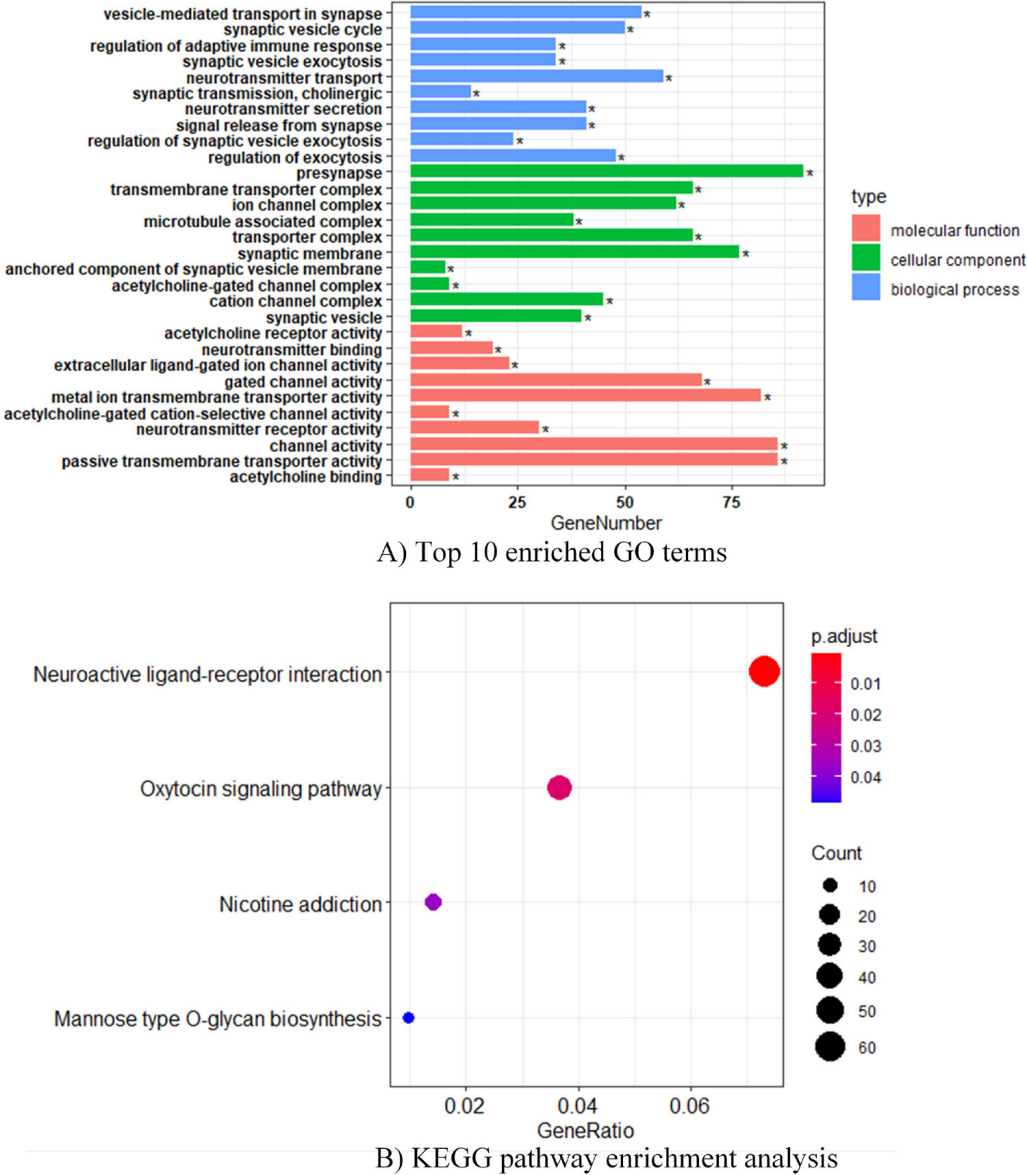
GO and KEGG enrichment analysis of HLA class II expression in SCLC. (A) Top 10 enriched GO terms in molecular function, cellular components, and biological processes. (B) KEGG pathway enrichment analysis. GO, gene ontology; HLA class II, human leukocyte antigen class II; KEGG, Kyoto Encyclopedia of Genes and Genomes; SCLC, small cell lung cancer.

To further investigate the biological pathways associated with HLA class II expression, we accomplished GO and KEGG enrichment analysis in SCLC patients in the GSE43346 dataset. As for GO terms enrichment, we exhibited respective top 10 enriched GO terms in MF, CC, and BP. HLA class II expression-related genes were prominently involved in presynapse (CC), synaptic membrane (CC), metal ion transmembrane transporter activity (MF), channel activity (MF), and passive transmembrane transporter activity (MF) (figure 3A). Besides, KEGG pathway analysis revealed that targeted genes were mainly clustered in the neuroactive ligand–receptor interaction signaling pathway (figure 3B).

### Landscape of immune infiltration in high-risk versus low-risk groups

**Figure 4:**
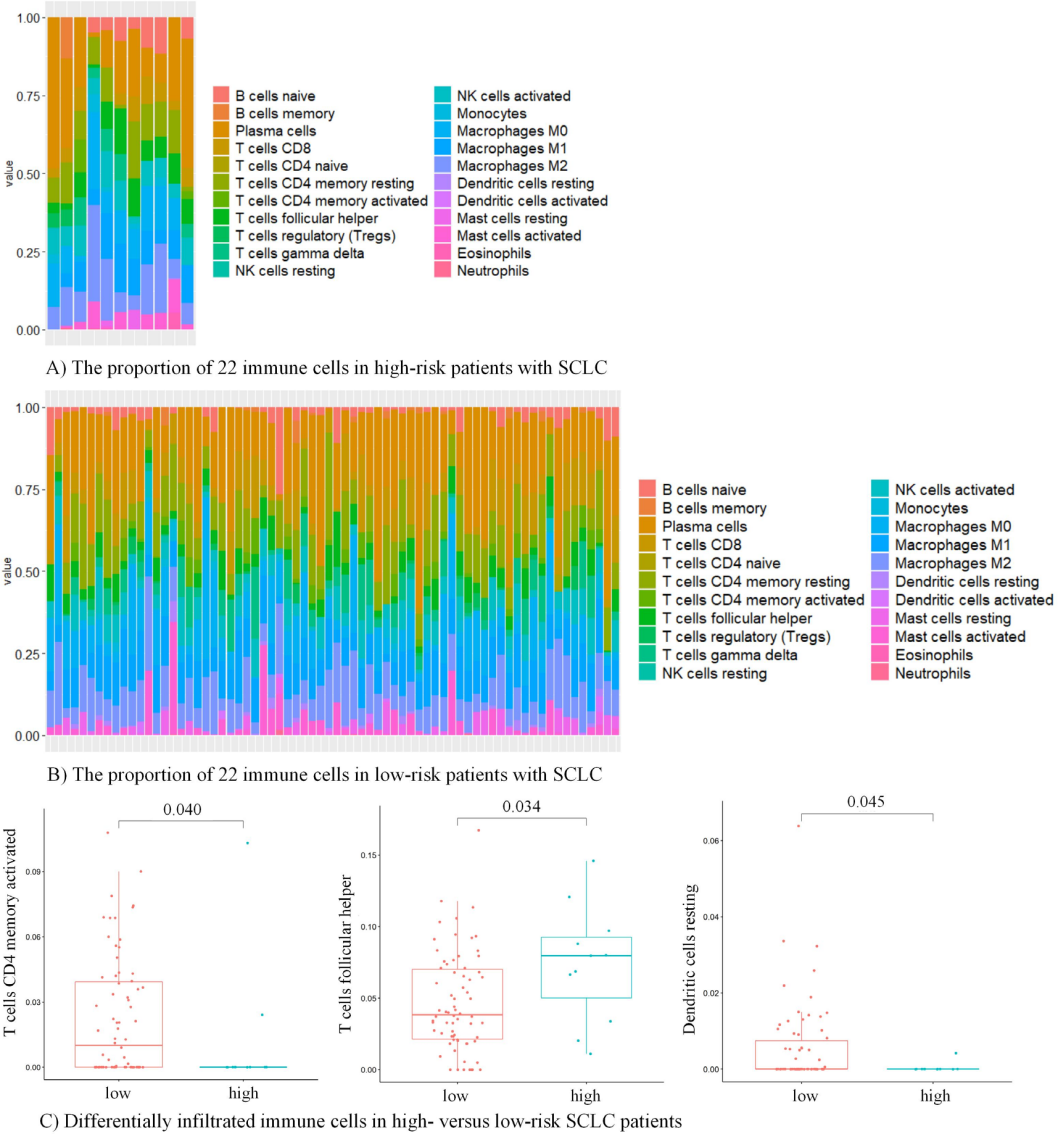
Immune infiltration in SCLC patients with high-risk and low-risk. (A) The proportion of 22 immune cells in high-risk patients with SCLC. (B) The proportion of 22 immune cells in low-risk patients with SCLC. (C) Differentially infiltrated immune cells in high-risk and low-risk patients with SCLC, including activated memory CD4+ T cells, T follicular helper cells, and resting dendritic cells. SCLC, small cell lung cancer.

To further investigate the association of immune infiltration with immune risk score, we used CIBERSORT to assess the distribution and composition of 22 immune cells among patients with SCLC. Based on mRNA expression levels of HLA class II gene, CD4, FOXP3, and CD274 encoding PD-L1, all 81 patients were classified into high-risk versus low-risk groups. The proportion of each immune cell differed between samples (figure 4A, B). After comparing the proportion of each immune cell among high-risk versus low-risk groups, we found SCLCpatients with high risk had a higher proportion of T follicular helper cells (p=0.034) and a lower proportion of activated memory CD4+ T cells (p=0.040) and resting dendritic cells (p=0.045) versus those with low risk (figure 4C). And immune cells in the high-risk group seemed to be more closely connected in comparison with those in the low-risk group (online supplemental figure 4A, B).

10.1136/jitc-2021-002554.supp5 Supplementary data

## Discussion

Immunotherapy has improved the survival of patients with SCLC, but little is known about efficacious predictive factors, which highlights the urgent need for better immune biomarkers to optimize therapeutic options. Herein, we analyzed the expression pattern of HLA class II and its connection with tumor immune microenvironment and prognosis. We detected positive HLA class II on few SCLC cells and nearly half of TILs. HLA class II on TILs was negatively correlated with lymph node metastasis and positively related to multiple immune markers and a longer RFS. Additionally, we established an HLA class II-based immune risk model that could more effectively predict the recurrence than tumor stage in SCLC. Using pathway analysis, we found signatures of transmembrane transportation, channel activity, and neuroactive ligand–receptor interaction were enriched in the high HLA class II expression group. And high-risk and low-risk patients with SCLC markedly differed in T follicular helper cells, activated memory CD4+ T cells, and resting dendritic cells. These findings indicate HLA class II plays a crucial role in tumor immune microenvironment and prognostic evaluation in patients with SCLC.

In this study, we observed that HLA class II was positively expressed on tumor cells in only 8.8% and on TILs in 44.1% of SCLC patients. The expression frequency of HLA class II on tumor cells varied among different types of cancers. Severe deficiency of HLA class II expression on tumor cells was also documented in other SCLC studies,^21 32^ hepatocellular carcinomas,^33^ ductal breast cancers,^34^ and acute myeloid leukemia.^35^ In our previous study of 72 Caucasian SCLC samples, no HLA class II was detected on tumor cells.^21^ The reasons for loss of HLA class II expression on tumor cells mainly included structural alterations (eg, mutations or rearrangements in HLA class II alleles) and deregulation of HLA class II antigen presentation machinery components induced by the promoter polymorphism or hypermethylation of HLA class II regulator CIITA.^36^ IFN-γ played a crucial role in the regulation of HLA class II expression to ensure an immune response against tumor cells.^36 37^ In vitro studies in SCLC and hepatocellular carcinomas showed the absence of HLA class II expression could not be rescued by IFN-γ induction,^32 33^ which to some extent could be considered as a form of antitumor immune escape and might partly explain their refractory features. Nonetheless, HLA class II was detectable in multiple other cancer types including NSCLC (30%),^21^ primary melanomas (50%–60%),^38^ thyroid papillary carcinomas (≈50%),^39^ pancreatic cancer (>70%),^40^ and colorectal cancers (21%–56%).^41–43^ As for HLA class II on TILs, our previous results revealed that HLA class II was positively expressed in only 15.3% Caucasian patients with SCLC.^21^ In the previous study including patients with NSCLC and SCLC, 11 (15.3%) of 72 patients with SCLC were positive for HLA class II with a cut-off value of 80%, markedly higher than 25% in this study. Given that the cut-off value was decided by the survival analysis of another NSCLC cohort with prognosis data rather than SCLC patients, the results may be incomparable. Overall, the differences of HLA class II expression level on tumor cells or TILs among a wide variety of tumors may be attributable to different research design, patient population, mechanisms of oncogenesis, molecular phenotypes, tumor origin, and various cut-off values for positivity.

We further analyzed the association of HLA class II with clinical factors. We found HLA class II expression on TILs was negatively related to lymph node metastasis, which indicated its protective function. Multiple studies have demonstrated that HLA class I was associated with lymph node metastasis in lung cancer,^44^ gastric cancer,^22 45^ and colorectal cancer.^46^ However, data about the involvement of HLA class II in lymph node metastasis is limited. Ogoshi *et al*^22^ found HLA-DR2, HLA-DR4, and HLA-DR6 on TILs had an opposite relation to lymph node metastasis in different histological subtypes and stages of patients with gastric cancers. Paradoxical to our result, lymph node metastasis showed a positive correlation with HLA-DR4 on TILs from the blood in gastric cancer^47^ and HLA-DR on tumor cells in lung cancer.^48^ In terms of the reasons for these discrepancies, we consider different histological types or subtypes, cancer stage, and expression location of HLA class II may influence the relationship with lymph node metastasis. Our study showed SCLC patients with positive HLA class II expression on TILs had less lymph node metastasis. We speculate that better recognition of HLA class II by CD4+ T cells may lead to the elimination of tumor cells and the prevention of migration and invasion. Future study is warranted to verify it and explore potential mechanisms.

In our current research, HLA class II on TILs was coexpressed with PD-1/PD-L1 on TILs and CD3, CD4, CD8, and FOXP3 on immune cells. It indicates that HLA class II on TILs may have a close connection with multiple immune markers. On the one hand, Kitamura *et al*^49^ demonstrated that downregulation of HLA class II expression induced by interleukin-6/STAT3 signaling activation hampered dendritic cells activating effector T cells in colorectal cancer. The impairment of HLA class II on TILs could account for the immunosuppression in NSCLC.^50^ On the other hand, considerable evidence supported T cells expressing HLA class II could function as APCs, also called T-APCs, and antigen presentation mediated by T-APCs could induce apoptosis or clonal anergy in activated T cells or cytotoxicity in resting T cells.^20^ Correspondingly, HLA class II-specific monoclonal antibodies were developed to treat hematological malignancy.^51^ Additionally, Costantini *et al*^18^ found HLA-DR mediated signaling increased the expression of adhesion receptors and PD-L1 and the activation of the JAK/STAT3 pathway, and promoted progression, migration, and invasion of melanoma cells. Taken together, HLA class II played an important role in tumor immune microenvironment, oncogenesis, and tumor progression. But it remains to be elucidated whether this association reflects the attraction of TILs by HLA class II or the upregulation of HLA class II-induced by IFN-γ secreted by TILs.

We also found HLA class II expressed on tumor cells did not correlate with clinical and other immune factors except for PD-1 on TILs. Similar results of insignificant correlation between HLA class II on tumor cells with CD4/CD8+ T cells was observed in intrahepatic cholangiocarcinoma.^52^ However, a significant association was observed between HLA class II expression and multiple immune molecules and cells, including PD-1/PD-L1 and CD8+ TILs in melanoma cells,^18 53^ CD4+ TILs in lymphomas and colorectal cancers,^54 55^ CD3+ TILs in mismatch repair-deficient cancers,^56^ and CD68+ and CD163+ tumor-associated macrophages in oropharyngeal cancer.^57^ As for SCLC, HLA class II on tumor cells had a limited association with immune markers, which might be ascribed to the extremely low level of HLA class II expression on tumor cells in SCLC in comparison with other types of cancers. Future larger sample-size clinical researches or fundamental investigation may further ascertain their association and potential mechanisms.

We observed RFS had no significant difference in SCLC patients with different expression status of HLA class II on tumor cells, and its rare expression limited further analysis. Nonetheless, RFS was significantly longer in SCLC patients positive for HLA class II on TILs as opposed to those negative, in accordance with our previous study.^21^ This favorable prognostic connection is not unique to SCLC, as it has recently been reported in a variety of solid and hematological tumors.^39 57–60^ In lymphoma and melanoma, HLA class II positivity on tumor cells was not only a favorable prognostic predictor but also an efficacious predictor of PD-1 blockade, thus producing the hypothesis of HLA class II-limited, CD4+ TILs-mediated action mechanism of PD-1 blockade.^58 60 61^ Additionally, the association of HLA class II with immune factors may partly account for its prognostic implication. Despite a lot of evidence supporting the favorable prognostic implications of positive HLA class II, other studies showed a discrepant correlation. HLA class II antigen was related to a poor prognosis in hepatocellular carcinoma and osteosarcomas.^62 63^ The inverse results may be induced by different oncogenesis mechanisms and heterogeneity of HLA class II subtypes. Overall, HLA class II has a definite link to RFS in SCLC, and its prognostic significance may be partly attributed to a close immune interaction.

Considering the close connection between HLA class II and immune markers and prognosis, we performed LASSO regression analysis among multiple immune variables to further analyze the influential factors of RFS in SCLC. And then we determined an HLA class II-based optimal risk model that categorized patients into high-risk and low-risk groups. High-risk patients with SCLC had a poorer prognosis, which was further confirmed in the public dataset. To our knowledge, it is the first time that we build an HLA class II-based immune risk model for prognostic evaluation in patients with SCLC, and it even has better predictive power than tumor stage.

In the final immune risk model, PD-L1, CD4, and FOXP3 were protective factors of RFS in patients with SCLC. PD-L1 on tumor cells or TILs was higher among early-stage SCLC compared with metastatic patients,^64^ and multiple authors demonstrated an association with better outcome in SCLC.^64–68^ However, limited studies reported contrary results.^69 70^ Different antibodies, cut-off values, and study design may cause the discrepancy. In our study, CD4+ TILs were closely associated with HLA class II and better prognosis in SCLC. CD4+ TILs recognition of HLA class II-restricted antigen could activate antitumor immunity.^11^ CD4+ T cells activation promoted differentiation into effector T cells and regulatory T (Treg) cells. The number of effector T cells in LS-SCLC was significantly more than that in ES-SCLC, and the high ratio of effector T cells to Treg cells predicted a long-term survival.^71^ FOXP3 is a specific biomarker of Treg cells, and its role in cancers is conflicting.^72^ Especially for SCLC, FOXP3+TILs presented inconsistent associations with prognosis. In a retrospective SCLC study, FOXP3+TILs were an independent positive prognostic factor in patients with non-metastatic SCLC at stage I–III, but not in metastatic patients.^64^ Our study also exhibited a positive association of FOXP3 with prognosis among patients with surgically resected SCLC at stage I–III. However, Kasahara *et al*^73^ found an insignificant correlation between FOXP3 and OS in patients with both LS-SCLC and ES-SCLC. The authors determined the cut-off value of FOXP3 based on survival analysis, but no specific value was provided. As for patients with metastatic SCLC, a similar insignificant association with survival was observed in another study that performed FOXP3+TILs IHC staining on brain metastasis specimens of patients with SCLC.^65^ Wang *et al*^74^ included 65 SCLC patients without stage information and demonstrated patients with a higher ratio of FOXP3/CD45 cells (measured as Tregs) had a worse median OS (120 vs 410 days). Considering its short OS, it may mainly consist of metastatic patients. The heterogeneous association of FOXP3 with prognosis in SCLC may be partly attributable to non-uniform cut-off values, tumor stage, and detection locations (primary vs metastatic). Additionally, FOXP3-positive T cells were constituted by three subpopulations: CD45RA(+)FOXP3(lo), CD45RA(−)FOXP3(hi), and CD45RA(−)FOXP3(lo).^75^ The former two were suppressive, and the third was non-suppressive T cells characterized by FOXP3 instability and inflammatory cytokines secretion. An abundance of inflammatory CD45RA(−)FOXP3(lo) T cells was correlated with a better prognosis in colorectal cancer.^76^ Therefore, the distinct predominant subpopulations of FOXP3+TILs during the dynamic process of tumor formation and progression may account for the different influences on prognosis in SCLC.

Furthermore, with bioinformatics methods, we investigated the implications of HLA class II and the immune risk model composed of HLA class II, PD-L1, CD4, and FOXP3 in immune infiltration and prognosis. After observing those SCLC patients with high HLA class II expression had a better prognosis versus low-expressed patients, we first performed GO and KEGG enrichment analyses to explore the potential biological pathways associated with HLA class II. Enriched pathways in the high-expression group involved transmembrane transport, channel activity, and ligand–receptor interaction. It indicates that HLA class II expression is associated with signaling transduction, as demonstrated in previous studies where ligation of HLA class II could transduce multiple intracellular signals such as protein kinase C membrane translocation and promote calcium influx.^20 77^ Then we did CIBERSORT analysis to delineate the immune infiltration landscape among high-risk and low-risk patients with SCLC. We found high-risk patients seemed to possess a closer interaction with immune cells and had significantly lower proportions of activated memory CD4+ T cells and resting dendritic cells versus those with low-risk. These differentially infiltrated immune cells correlated closely with antigen presentation and immune response. Collectively, we demonstrated a close relationship between HLA class II molecules, tumor immune microenvironment, and prognosis. Multiple studies mentioned that, except for the well-established role of tumor neoantigen-specific CD8+ T cells in antitumor immunity, HLA class II presenting neoantigens to CD4+ T cells also exerted a crucial function in tumor eradication and response to immunotherapy.^58 78 79^ Despite that, some cancer patients responded to immunotherapy even without detectable HLA class II,^58^ indicating that the mechanisms of HLA class II in immune regulation and antitumor immunity remain to be elaborated.

Finally, we acknowledge several limitations in our research. First of all, lack of representativeness may present as this study is single-center and retrospective. Second, given that there are no clear and standard cut-off values for HLA class II and multiple immune indicators, we determined the optimal cut-off values for immune markers by survival analysis. The repeatability and clinical utility of the optimal cut-off values need more studies to verify. Third, we established an immune risk model after cross-validation in the LASSO regression. Further validation and refinement are warranted in the future studies. Finally, not enough long follow-up duration restrains from comparing OS, but RFS already showed a sufficiently significant difference.

## Conclusion

In this study, we detected positive HLA class II on few SCLC cells and nearly half of TILs. And we found positivity of HLA class II on TILs was negatively associated with lymph node metastasis and positively related to multiple immune markers and a better RFS. Furthermore, we established an HLA class II-based immune risk model, which can not only more effectively predict the recurrence than tumor stage but also identify immune infiltration heterogeneity in high-risk and low-risk patients with SCLC. Taken together, these findings indicate that HLA class II plays a crucial role in tumor immune microenvironment and prognostic evaluation in SCLC patients. Future studies are warranted to reveal the mechanism behind it.

## Data Availability

Data are available upon reasonable request. All data relevant to the study are included in the article or uploaded as supplementary information. Data are available upon reasonable request. All data relevant to this study are included in the article or online supplemental materials.
