# Supplementary figures and images for: Human leukocyte antigen class II-based immune risk model for recurrence evaluation in stage I–III small cell lung cancer
Source: J Immunother Cancer. 2021 Aug 6;9(8):e002554. doi: 10.1136/jitc-2021-002554 (PMC8351500; doi:10.1136/jitc-2021-002554)

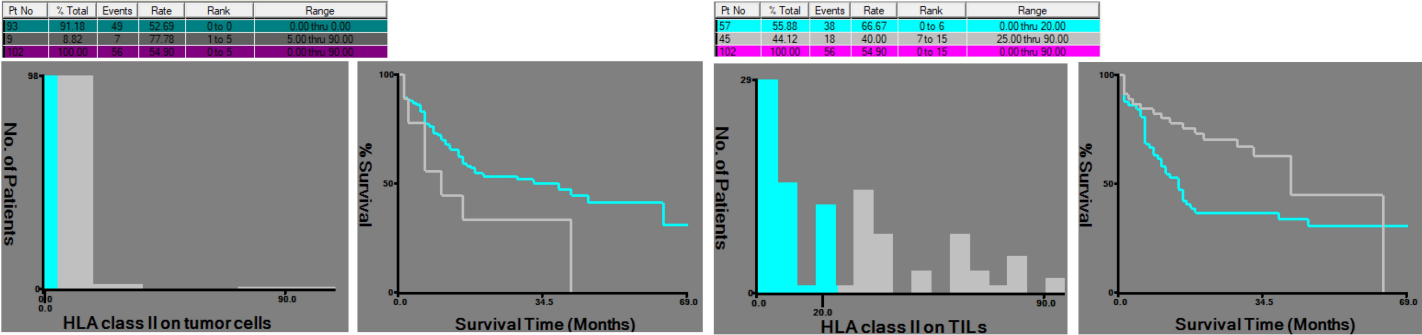

A) The cut-off value of HLA class II on tumor cells

B) The cut-off value of HLA class II on TILs

Supplement: Supplementary data [file jitc-2021-002554supp002.pdf]
