# Supplementary material for: Human leukocyte antigen class II-based immune risk model for recurrence evaluation in stage I–III small cell lung cancer
Source: J Immunother Cancer. 2021 Aug 6;9(8):e002554. doi: 10.1136/jitc-2021-002554 (PMC8351500; doi:10.1136/jitc-2021-002554)
Supplement: Supplementary data [file jitc-2021-002554supp001.pdf]

BMJ Publishing Group Limited (BMJ) disclaims all liability and responsibility arising from any reliance placed on this supplemental material which has been supplied by the author(s)

Table S1 Characteristics of 102 SCLC patients

Sample ID	Age, years	Sex	Smoke	Stage	Pathology review	HLA II expression on TILs, %	HLA II expression on tumor cells, %
1	55	male	non-smoker	1	SCLC	40	0
2	52	male	non-smoker	3	SCLC	30	0
3	76	male	non-smoker	1	SCLC	90	0
4	58	male	non-smoker	2	SCLC	20	0
5	55	male	non-smoker	1	SCLC	10	0
6	51	male	non-smoker	3	SCLC	5	0
7	76	male	smoker	3	SCLC	10	10
8	47	female	non-smoker	3	SCLC	0	0
9	65	female	non-smoker	3	SCLC	5	0
10	72	male	non-smoker	3	SCLC	5	0
11	73	male	non-smoker	3	SCLC	2	0
12	56	male	non-smoker	3	SCLC	0	0
13	66	male	smoker	3	SCLC	40	0
14	58	male	smoker	3	SCLC	1	0
15	68	female	non-smoker	3	SCLC	60	20
16	60	male	non-smoker	2	SCLC	15	0
17	67	male	non-smoker	2	SCLC	0	0
18	43	female	non-smoker	2	SCLC	90	0
19	76	male	non-smoker	3	SCLC	20	0
20	53	male	non-smoker	1	SCLC	30	0
21	60	male	smoker	3	SCLC	30	0
22	51	female	non-smoker	3	SCLC	5	0
23	52	male	non-smoker	3	SCLC	10	5
24	78	male	non-smoker	1	SCLC	20	0
25	54	male	non-smoker	3	SCLC	20	0
26	74	male	non-smoker	3	SCLC	5	0
27	60	male	non-smoker	2	SCLC	10	0
28	68	female	non-smoker	3	SCLC	0	0
29	66	female	non-smoker	2	SCLC	1	0
30	73	male	non-smoker	1	SCLC	10	0
31	65	male	non-smoker	2	SCLC	5	0
32	67	male	smoker	1	SCLC	80	0
33	73	female	non-smoker	3	SCLC	10	0
34	75	male	smoker	1	SCLC	80	20
35	59	male	smoker	2	SCLC	60	0
36	75	male	smoker	1	SCLC	20	0
37	65	male	smoker	1	SCLC	20	0
38	63	female	non-smoker	1	SCLC	60	0

Chen P, *et al. J Immunother Cancer* 2021; 9:e002554. doi: 10.1136/jitc-2021-002554

39	57	male	smoker	3	SCLC	30	0
40	40	male	non-smoker	1	SCLC	20	0
41	42	male	smoker	2	SCLC	0	0
42	58	male	non-smoker	2	SCLC	1	0
43	54	male	smoker	3	SCLC	30	0
44	72	male	smoker	3	SCLC	5	0
45	56	female	non-smoker	2	SCLC	10	0
46	66	male	smoker	1	SCLC	50	0
47	70	male	smoker	3	SCLC	10	0
48	52	male	smoker	3	SCLC	60	0
49	66	male	smoker	3	SCLC	20	0
50	77	male	smoker	3	SCLC	60	0
51	63	female	non-smoker	3	SCLC	5	0
52	74	male	smoker	3	SCLC	40	0
53	64	male	smoker	2	SCLC	40	0
54	67	male	non-smoker	3	SCLC	30	0
55	81	male	smoker	1	SCLC	30	0
56	38	male	non-smoker	1	SCLC	30	0
57	63	male	smoker	2	SCLC	30	0
58	76	male	non-smoker	3	SCLC	20	0
59	67	male	smoker	3	SCLC	80	0
60	74	male	smoker	3	SCLC	5	0
61	67	female	non-smoker	1	SCLC	1	0
62	63	male	non-smoker	3	SCLC	10	0
63	47	female	non-smoker	2	SCLC	0	90
64	66	male	smoker	2	SCLC	10	10
65	68	male	non-smoker	1	SCLC	30	0
66	61	male	non-smoker	1	SCLC	40	0
67	72	male	smoker	1	SCLC	30	0
68	68	male	smoker	1	SCLC	30	0
69	61	female	non-smoker	1	SCLC	50	80
70	49	male	smoker	3	SCLC	0	0
71	68	male	non-smoker	3	SCLC	40	0
72	54	male	non-smoker	2	SCLC	20	0
73	52	male	non-smoker	1	SCLC	5	0
74	58	male	smoker	3	SCLC	10	0
75	63	male	smoker	3	SCLC	1	0
76	64	male	smoker	2	SCLC	10	0
77	51	male	smoker	2	SCLC	40	0
78	61	male	smoker	1	SCLC	50	0
79	68	male	non-smoker	3	SCLC	70	0
80	62	male	smoker	2	SCLC	20	0

Chen P, *et al. J Immunother Cancer* 2021; 9:e002554. doi: 10.1136/jitc-2021-002554

81	63	female	non-smoker	3	SCLC	60	0
82	55	male	non-smoker	3	SCLC	10	0
83	66	male	smoker	1	SCLC	0	5
84	73	male	smoker	2	SCLC	5	0
85	63	male	non-smoker	1	SCLC	70	0
86	65	male	non-smoker	1	SCLC	40	0
87	69	male	smoker	1	SCLC	70	0
88	42	male	non-smoker	3	SCLC	10	0
89	70	female	non-smoker	1	SCLC	5	0
90	51	male	non-smoker	1	SCLC	80	0
91	60	male	non-smoker	2	SCLC	60	0
92	68	male	smoker	3	SCLC	20	0
93	66	male	smoker	1	SCLC	75	0
94	72	male	smoker	1	SCLC	1	0
95	67	male	smoker	1	SCLC	60	0
96	63	female	non-smoker	1	SCLC	25	0
97	63	male	non-smoker	2	SCLC	5	0
98	78	male	smoker	1	SCLC	30	0
99	51	male	smoker	1	SCLC	30	0
100	69	male	smoker	1	SCLC	10	0
101	62	male	non-smoker	1	SCLC	5	0
102	61	female	non-smoker	1	SCLC	80	5

HLA II, human leukocyte antigen class II; SCLC, small cell lung cancer; TILs, tumor infiltrating lymphocytes.

Table S2 Correlation between HLA class II and other immune factors

Biomarkers	HLA class II on tumor cells		HLA class II on TILs	
	Correlation coefficient	P value	Correlation coefficient	P value
HLA class II on tumor cells	NA	NA	0.028	0.780
HLA class II on TILs	0.028	0.780	NA	NA
PD-1 on TILs	0.273	0.006*	0.410	<0.001*
PD-L1 on TILs	0.063	0.527	0.511	<0.001*
PD-L1 on tumor cells	-0.063	0.531	0.106	0.288
CD3	0.140	0.161	0.441	<0.001*
CD4	0.128	0.199	0.582	<0.001*
CD8	0.151	0.130	0.496	<0.001*
FOXP3	0.123	0.219	0.507	<0.001*

*, P<0.05 indicates statistical significance; NA, not available; HLA class II, human leukocyte antigen class II; PD-1, programmed death-1; PD-L1, programmed death-ligand 1; TILs, tumor infiltrating lymphocytes

Chen P, *et al. J Immunother Cancer* 2021; 9:e002554. doi: 10.1136/jitc-2021-002554

Table S3 Bivariate logistic regression of HLA class II expression on TILs

Variables	Univariate			Multivariate		
	OR	95% CI	P value	OR	95% CI	P value
Age (<70 vs. ≥70)	0.472	0.175-1.273	0.138			
Gender (female vs. male)	0.770	0.272-2.180	0.623			
Smoking status (non-smoker vs. smoker)	0.473	0.213-1.052	0.066			
T (1–2 vs. 3–4)	0.268	0.071-1.016	0.053			
N (0 vs. 1–2)	0.283	0.124-0.645	0.003*	0.294	0.108-0.799	0.016*
M (0 vs. 1)	1.279	0.173-9.453	0.809			
Stage (I vs. II-III)	0.468	0.207-1.059	0.068			
Adjuvant chemotherapy (no vs. yes)	1.140	0.521-2.496	0.742			
PD-1 on TILs (negative vs. positive)	2.689	1.19-6.078	0.017*	0.557	0.160-1.937	0.357
PD-L1 on TILs (negative vs. positive)	7.741	3.114-19.242	<0.001*	3.339	1.071-10.404	0.038*
PD-L1 on tumor cells (negative vs. positive)	4.000	0.402-39.827	0.237			
CD3 (negative vs. positive)	7.000	2.866-17.094	<0.001*	3.309	0.891-12.281	0.074
CD4 (negative vs. positive)	5.132	2.152-12.238	<0.001*	1.031	0.268-3.968	0.965
CD8 (negative vs. positive)	6.095	2.425-15.323	<0.001*	1.157	0.298-4.493	0.833
FOXP3 (negative vs. positive)	9.187	3.531-23.903	<0.001*	3.021	0.668-13.669	0.151

*, P<0.05 indicates statistical significance; OR, Odds ratio; 95% CI, 95% confidence interval; HLA class II, human leukocyte antigen class II; PD-1, program death-1; PD-L1, program death-ligand 1; TILs, tumor infiltrating lymphocytes.
