# Supplementary material for: Human leukocyte antigen class II-based immune risk model for recurrence evaluation in stage I–III small cell lung cancer
Source: J Immunother Cancer. 2021 Aug 6;9(8):e002554. doi: 10.1136/jitc-2021-002554 (PMC8351500; doi:10.1136/jitc-2021-002554)
Supplement: Supplementary data [file jitc-2021-002554supp004.pdf]

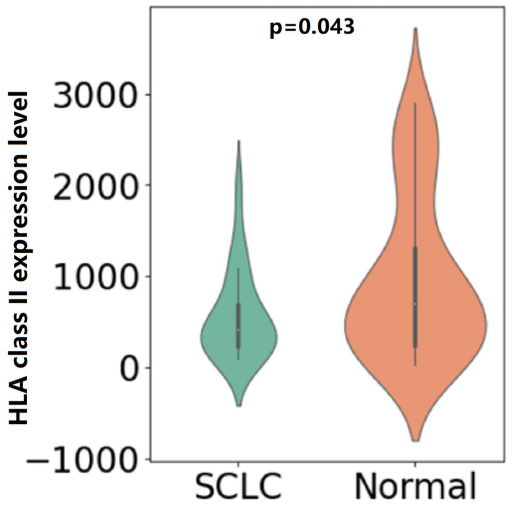

A) Total HLA class II expressed in SCLC patients versus normal tissues

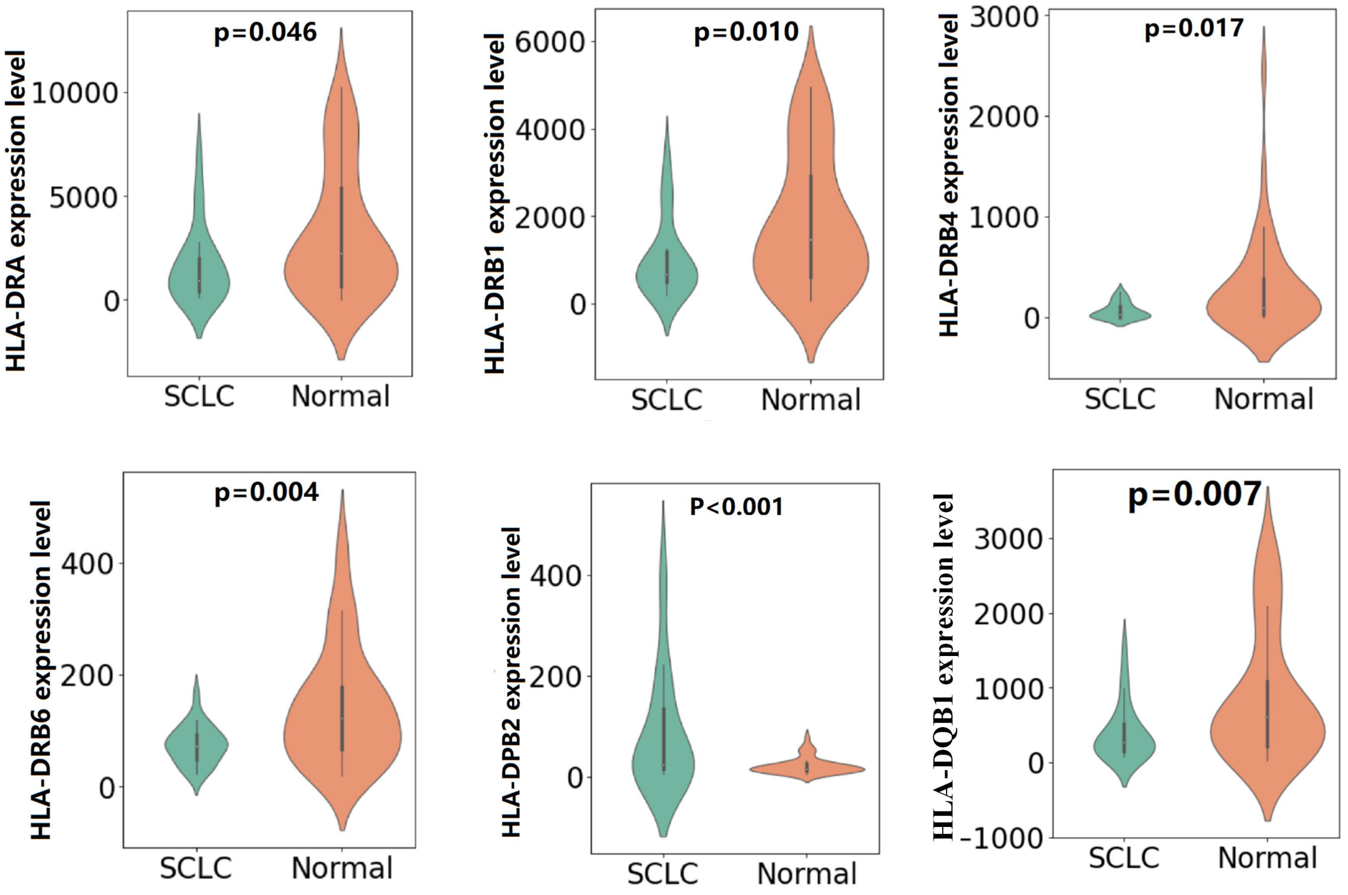

B) Differentially expressed HLA class II subtypes in SCLC patients versus normal tissues

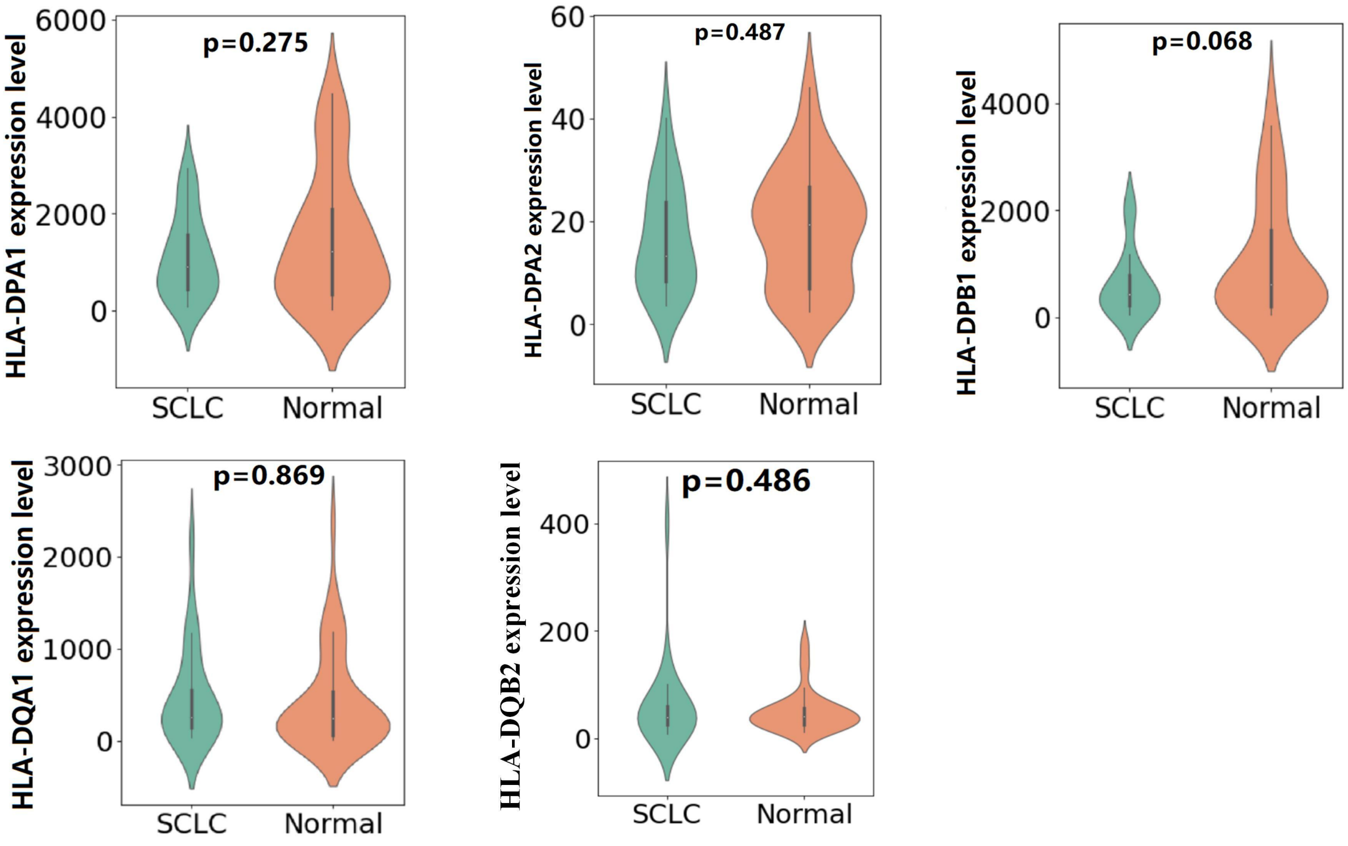

C) Non-differentially expressed HLA class II subtypes in SCLC patients versus normal tissues
